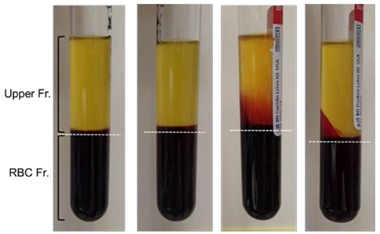# An Evaluation of the Accuracy of the Subtraction Method Used for Determining Platelet Counts in Advanced Platelet-Rich Fibrin and Concentrated Growth Factor Preparations

**DOI:** 10.3390/dj5010007

**Published:** 2017-01-12

**Authors:** Taisuke Watanabe, Kazushige Isobe, Taiji Suzuki, Hideo Kawabata, Masayuki Nakamura, Tsuneyuki Tsukioka, Toshimitsu Okudera, Hajime Okudera, Kohya Uematsu, Kazuhiro Okuda, Koh Nakata, Tomoyuki Kawase

**Affiliations:** 1 Tokyo Plastic Dental Society, Kita-ku, Tokyo 114-0002, Japan; watatai@mui.biglobe.ne.jp (T.W.); kaz-iso@tc4.so-net.ne.jp (K.I.); drtaiji1116@yahoo.co.jp (T.S.); hidei@eos.ocn.ne.jp (H.K.); maoh4618@me.com (M.N.); ugk64590@nifty.com (T.T.); toshiokuderaphd@gmail.com (T.O.); okudera@carrot.ocn.ne.jp (H.O.); 2 Division of Implantology, Niigata University Medical and Dental Hospital, Niigata 951-8514, Japan; ue@dent.niigata-u.ac.jp; 3 Division of Periodontology, Institute of Medicine and Dentistry, Niigata University, Niigata 951-8514, Japan; okuda@dent.niigata-u.ac.jp; 4 Bioscience Medical Research Center, Niigata University Medical and Dental Hospital, Niigata 951-8520, Japan; radical@med.niigata-u.ac.jp; 5 Division of Oral Bioengineering, Institute of Medicine and Dentistry, Niigata University, Niigata 951-8514, Japan

**Keywords:** fractionation, platelets, platelet-rich fibrin, concentrated growth factors, quality assurance, regenerative therapy

## Abstract

Platelet concentrates should be quality-assured of purity and identity prior to clinical use. Unlike for the liquid form of platelet-rich plasma, platelet counts cannot be directly determined in solid fibrin clots and are instead calculated by subtracting the counts in other liquid or semi-clotted fractions from those in whole blood samples. Having long suspected the validity of this method, we herein examined the possible loss of platelets in the preparation process. Blood samples collected from healthy male donors were immediately centrifuged for advanced platelet-rich fibrin (A-PRF) and concentrated growth factors (CGF) according to recommended centrifugal protocols. Blood cells in liquid and semi-clotted fractions were directly counted. Platelets aggregated on clot surfaces were observed by scanning electron microscopy. A higher centrifugal force increased the numbers of platelets and platelet aggregates in the liquid red blood cell fraction and the semi-clotted red thrombus in the presence and absence of the anticoagulant, respectively. Nevertheless, the calculated platelet counts in A-PRF/CGF preparations were much higher than expected, rendering the currently accepted subtraction method inaccurate for determining platelet counts in fibrin clots. To ensure the quality of solid types of platelet concentrates chairside in a timely manner, a simple and accurate platelet-counting method should be developed immediately.

## 1. Introduction

Ever since platelet-rich plasma (PRP) was reported to be effective for skeletal regeneration in sinus floor elevation [1], PRP and its other subsequently developed derivatives, all of which can be designated as “platelet concentrates”, have been widely applied as a source of growth factors in various fields of regenerative therapy. In the fields of periodontology and maxillofacial surgery, platelet-rich fibrin (PRF), a PRP derivative, has increasingly been used for treatment of hard and soft tissues [2,3,4,5]. In terms of their preparation, the principle behind the fractionation of blood cell components remains misunderstood and actual counts of the components may be overestimated. Centrifugal fractionation is the most efficient method available for separating particles of different specific gravities and sizes. However, because blood cells are not ideally spherical or mechanically stiff, they cannot be clearly fractionated according to their specific gravity and size. Among most clinicians involved in regenerative therapy, it has nevertheless been generally accepted that platelets are highly concentrated in the buffy coat and are hardly present in other neighboring fractions, especially the red blood cell (RBC) fraction, after centrifugal fractionation.

This misunderstanding is not limited to the efficiency of evaluating platelet concentrations in liquid samples but can be expanded to the evaluation of platelet counts in self-clotted platelet concentrates, such as advanced-platelet-rich fibrin (A-PRF) and concentrated growth factors (CGFs). Since platelets have no nuclei, their counts cannot be determined by DNA contents. Therefore, to determine platelet counts in fibrin clots, a “subtraction method” is currently applied for the calculation [6,7,8,9]. According to this method, the platelet counts contained in fibrin clots are calculated by subtracting those in the clot exudate, the supernatant serum, and the RBC fraction (i.e., the red thrombus) from those in the starting whole blood sample. However, this method does not consider the possibility of platelet contamination in the RBC fraction or the possible loss and damage of platelets during processing for cell counting.

Therefore, we have long suspected the validity of this method. To assure the quality of platelet concentrates, the purity and identity, at least, should be evaluated in each preparation prior to clinical use, as described in the guidelines for major advanced therapeutic medicinal products [10,11]. To evaluate the possible contamination and loss of platelets in the preparation process in self-clotted platelet concentrates, in this study, we examined the distribution of platelets and white blood cells (WBCs) after fractionation by the centrifugal protocols recommended for their preparation. From the results, we found that the subtraction method is not appropriate for the accurate quantification of platelets in fibrin clots.

## 2. Results

The appearances of the samples in the presence of the anti-coagulant after fractionation under the indicated centrifugal conditions are shown in Table 1. The buffy coat, a thin white line just above the RBC fraction, was somewhat more clearly formed by high-speed centrifugation using a swing rotor, which is recommended for the 1st spin of PRP preparation. In contrast, the low-speed centrifugation recommended for A-PRF preparation could not clearly fractionate RBCs, which were, to some extent, diffused into the upper plasma fraction.

**Figure 1:**
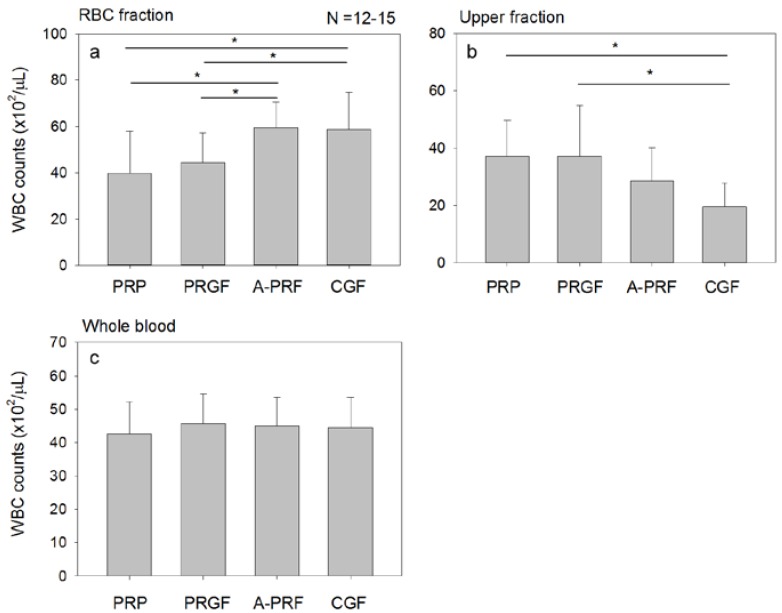
WBC counts in the RBC fraction (**a**), upper plasma fraction (**b**), and whole blood sample (**c**). Peripheral blood samples were collected in the presence of the anti-coagulant and centrifuged by the individual centrifugation protocols. The upper fraction was collected above the line indicated in Table 1. The remainder of the fractionated sample was used as the RBC fraction. N = 12–15. The asterisks represent statistically significant difference (*p* < 0.05).

The WBC distributions of the fractionated samples are shown in Figure 1. Whole blood samples containing the anti-coagulant were centrifuged by the indicated protocols as in Table 1. WBC counts (i.e., concentration) in the RBC fraction were significantly higher in the A-PRF/CGF-simulation models than in the PRP/plasma rich in growth factors (PRGF)-simulation models, whereas those in the upper fraction tended to be lower in the A-PRF/CGF-simulation models. The common factors of the A-PRF- and CGF-simulation models were the use of glass tubes and an angle rotor, but not the centrifugal force.

**Figure 2:**
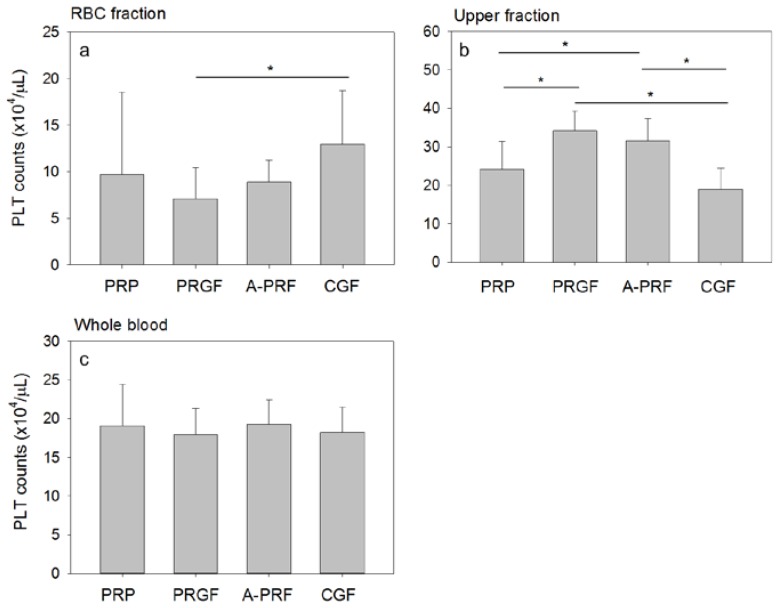
Platelet (PLT) counts in the RBC fraction (**a**), upper plasma fraction (**b**), and whole blood sample (**c**). Peripheral blood samples were collected in the presence of the anti-coagulant and centrifuged by the individual centrifugation protocols. The upper fraction was collected above the line indicated in Table 1. The rest of the fractionated sample was used as the RBC fraction. N = 12–15. The asterisks represent statistically significant difference (*p* < 0.05).

Platelet distributions in the fractionated samples are shown in Figure 2. Whole blood samples were fractionated as in Table 1. Platelet counts in the RBC fraction were relatively lower in the PRGF-simulation model. Instead, those in the upper fraction were significantly higher in both the PRGF- and the A-PRF-simulation models than that of the PRP- and CGF-simulation models. Both the PRGF- and A-PRF-simulation models adopted relatively low-speed centrifugation.

**Figure 3:**
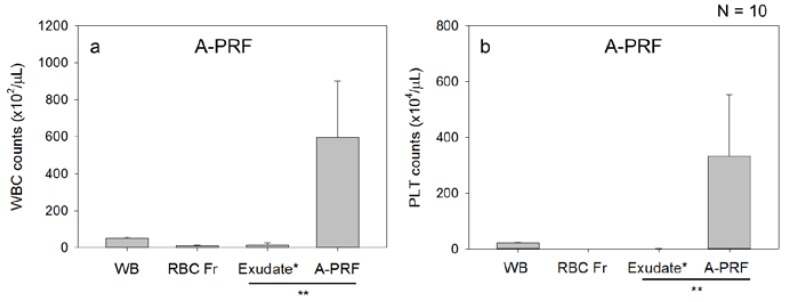

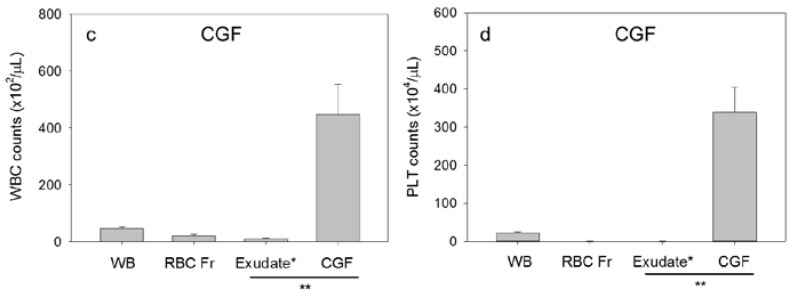
WBC (**a**,**c**) and platelet (PLT) counts (**b**,**d**) in the A-PRF (**a**,**b**) and CGF preparations (**c**,**d**). Peripheral blood samples were collected into glass tubes in the absence of the anti-coagulant and centrifuged by the individual centrifugation protocols. The freshly formed fibrin clots were withdrawn and dissected from the RBC clots. The resulting A-PRF/CGF preparations were compressed to collect the exudate fractions, whereas the RBC clots were minced and gently combined with the liquid form of RBC fraction. WBC and platelet counts were determined in the whole samples, A-PRF/CGF exudate fraction, and RBC fractions. Calculated WBC and platelet counts in the A-PRF/CGF preparations, which were calculated by the subtraction method, were substantially greater than that of the other fractions. N = 10. * The exudate fraction also included a small volume of the acellular serum fraction. ** Sum of these fractions corresponds to the upper plasma fraction shown in Table 1.

To demonstrate similarity, WBC and platelet distributions in the self-clotted A-PRF/CGF preparations were examined. As shown in Figure 3, in the A-PRF/CGF prepared from the blood samples collected in the absence of the anti-coagulant, both apparent WBC and platelet counts in the sum of the RBC and exudate fractions were substantially lower than those in the whole blood samples. As a result, the calculated WBC and platelet counts in the clotted A-PRF/CGF fractions were extraordinarily higher than those in the other fractions. These findings indicate that, unlike those in the upper fractions obtained in the presence of the anti-coagulant, both WBCs and platelets are evaluated to be concentrated predominantly in the A-PRF/CGF clots by the subtraction method according to the Formula (1) described in the Materials and Methods section.

**Figure 4:**
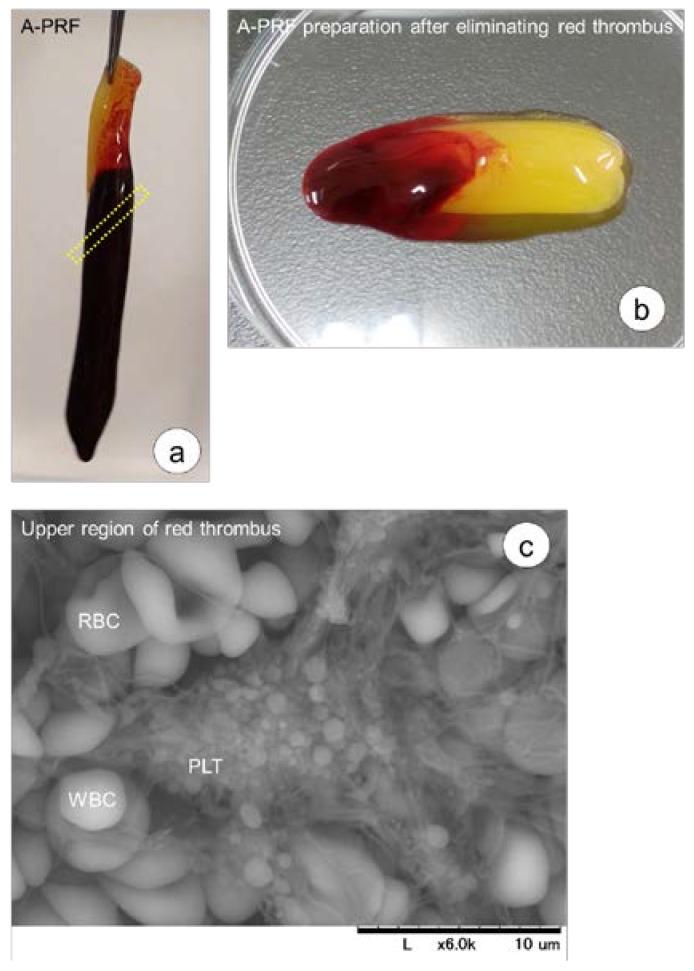
(**a**) Appearance of the A-PRF clot prepared using a centrifuge equipped with an angle rotor and the recommended glass tube. (**b**) The A-PRF preparation with a small portion of the red thrombus was prepared by scrapping off most of the red thrombus. (**c**) The region indicated by the yellow dot-square was subjected to examination by Scanning Electron Microscopy (SEM). In the CGF preparation, the red thrombus was usually shorter than that of the A-PRF preparation. SEM observations of aggregated platelets in the upper region of the RBC clot formed just below A-PRF preparations. Similar findings were obtained in the preparation of CGF. Bar = 10 μm.

To detect platelets in the red thrombus, the upper region of the red thrombus, indicated by the yellow dot square that is approximately 2 mm below the cutting edge in the upper left panel of Figure 4, was dissected and washed three times with PBS to remove RBCs loosely trapped by fibrin meshwork of the clot. Platelet aggregation in the upper region of the red thrombus is shown in the lower panel of Figure 4. In contrast to RBCs, WBCs and platelets seemed to tightly attach to fibrin fibers, and platelet aggregates were found almost everywhere on the surface of this region.

**Figure 5:**
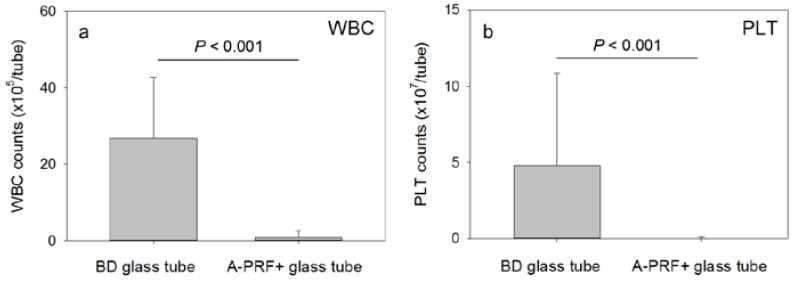
WBC (**a**) and platelet counts (**b**) on the inside wall of two types of glass tubes. After the A-PRF/CGF clots prepared in the absence of the anti-coagulant were removed, the inside of the tubes were washed thoroughly. WBCs and platelets were enzymatically detached for counting. N = 8 (A-PRF+^®^) or 12 (Vacutainer tube^®^).

The possible loss of platelets during collection and centrifugation was then examined. The number of WBCs and platelets that tightly adhered to the inside wall of the glass tubes is shown in Figure 5. In the glass tubes recommended for A-PRF preparations (A-PRF+^®^), as well as in plastic tubes (Neotube^®^) (data not shown), washing with PBS three times thoroughly removed almost all of the potentially attached WBCs and platelets. In contrast, a significant number of WBCs and platelets were detected in the glass tubes usually used for the preparation of CGF (Vacutainer tube^®^). These findings indicate that, possibly because of insufficient or absent surface siliconization or other coating, particular types of glass tubes, such as the Vacutainer tube^®^, allow WBCs and platelets to tightly adhere to their inside walls along with a more efficient induction of clotting.

**Figure 6:**
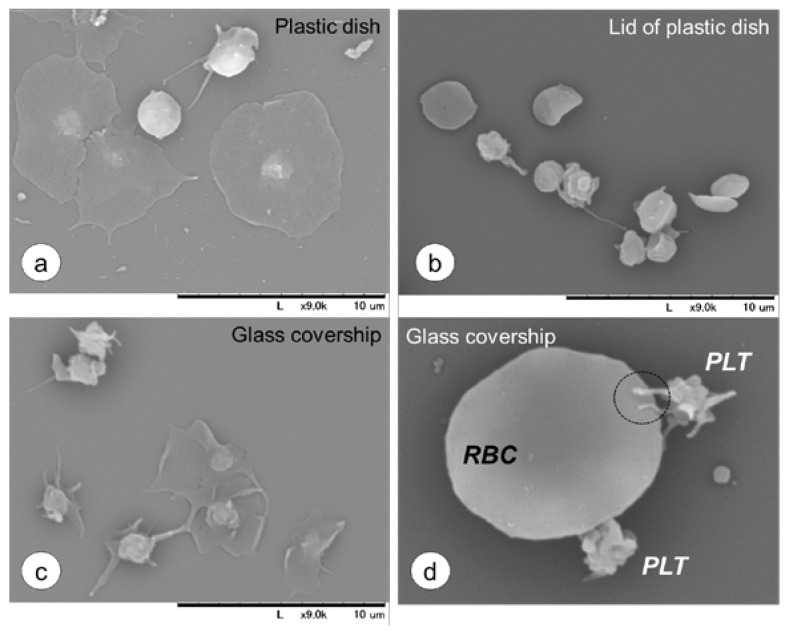
SEM observations of platelets on cell culture wares in the absence or the presence of RBCs. Washed platelets suspended in Hepes–Tyrode solution were placed on plastic dishes (**a**), hydrophobic lids of plastic dishes (**b**) or glass coverslips (**c**) and incubated for 10 min at 37 °C. As above, RBC-contaminated platelet suspensions were plated on the coverslip and incubated (d). Data is representative of three independent experiments. Bar = 10 μm.

To confirm this observation, the basic ability of platelet’s adhesion to plastic and glass labware and RBC was examined using SEM. As shown in Figure 6, platelets adhered to polystyrene culture dishes and glass coverslips within 10 min. Some platelets emitted pseudopodia, whereas others were spread to form flatten disks. In contrast, in the lid of a plastic culture dish that is not modified for better cell affinity, most platelets appeared in a resting state. Furthermore, in the presence of RBCs, some platelets were found to attach to RBCs by their pseudopodia, as indicated by the dotted-line circle.

## 3. Discussion

In general, as the centrifugal force increases, the volume of the RBC fraction becomes smaller, whereas that of the upper fraction becomes larger. It should be noted that this phenomenon by itself provides the background influencing blood cell counts, i.e., blood cell concentration, in each fraction. Under these conditions, WBC counts did not appear to be significantly influenced by the centrifugal force. Centrifugation using an angle rotor and glass tubes, regardless of centrifugal force, significantly increased WBC counts in the RBC fraction, whereas the WBC counts in the upper plasma fraction were decreased. In contrast, platelet counts were apparently influenced by centrifugal force; they were increased in the upper plasma fraction by low speed centrifugation, whereas those in the RBC fraction were decreased.

The main purpose of this study was to validate the currently accepted method of determining platelet counts in fibrin clots. The current method involves subtracting the platelet counts in the RBC, supernatant “acellular” serum, and A-PRF/CGF exudate fractions from those in the whole blood sample [6,7,8,9]. However, given that platelets easily aggregate upon activation and adhere to glass/plastic/metal surfaces, we have suspected that platelet counts can be determined accurately by cell counting in the liquid/semi-clotted fractions after multiple steps and subsequent calculation.

In this study, we demonstrated that platelets easily and rapidly attach to both glass and plastic surface optimized for cell adhesion. However, platelets appeared not to attach to hydrophobic plastic surface. Judging from the disclosed manufacturers’ information and research investigators’ data [12], it is thought that the inside wall of blood collection tubes is generally, but not invariably, coated with silicon or similar agents to prevent cell adhesion. Therefore, possible platelet adhesion to the inside wall of the tube may be low and insignificant. On the other hand, loss by platelet adhesion to the stainless-steel compression device or dry gauze and damage during compression to squeeze exudates should be considered as demonstrated in the previous study [13].

**Figure 7:**
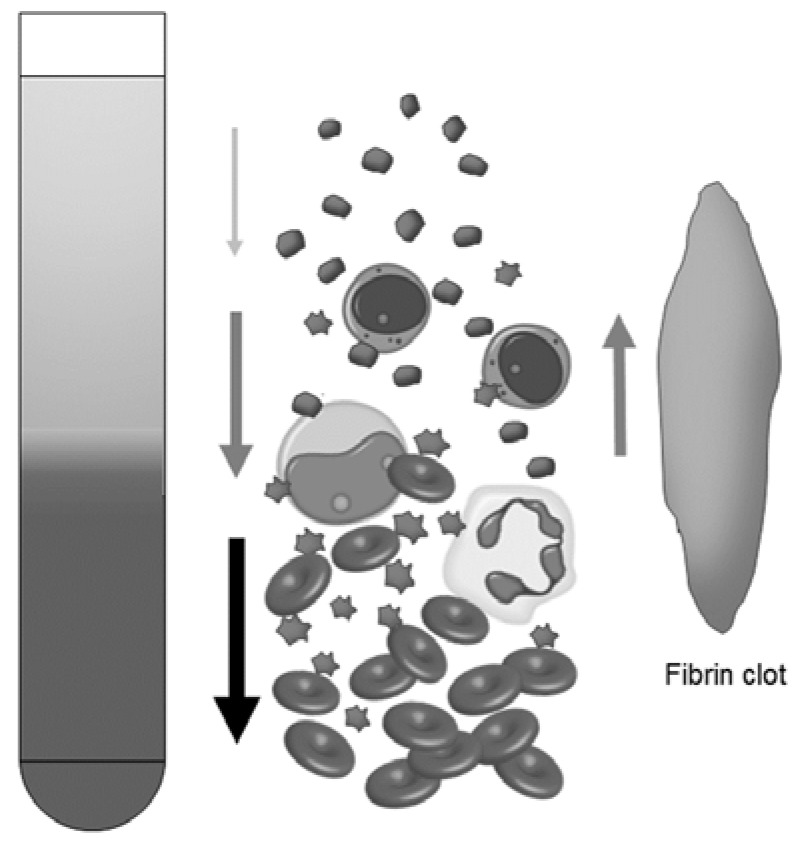
Scheme of migration of major blood components during centrifugation.

However, we would indicate that the contamination of platelets in the RBC fraction, i.e., the red thrombus is the major factor causing miscalculation of platelet counts. The scheme of migration of major blood components during centrifugation is illustrated in Figure 7. Owing to their deformability and Fe^2+^-dependent high specific gravity, although smaller in size than large WBCs, RBCs spin-down faster. Specific binding and mechanical interaction, to some extent, cause RBCs to bring platelets into the RBC fractions, as demonstrated in previous studies [14,15]. WBCs and platelets spin-down essentially depending on their size. However, if aggregated, platelets would spin-down faster than WBCs. Conversely, the fibrin clot captures blood cells, especially platelets and WBCs, and tends to rise to the upper surface by buoyant force. These reactions are synchronized to form A-PRF/CGF clots.

To release possibly contaminating platelets and WBCs and determine their counts more accurately, we did mince the red thrombus using scissors; however, the resulting platelet counts were much lower than those in the RBC fraction in the presence of the anti-coagulant. Taken together with the finding that platelet aggregation was actually detected in the red thrombus, it should be noted that a significant number of platelets is included in the red thrombus and isolated from subsequent calculation.

As for possible alternatives, the measurement of PDGF, a major growth factor produced in platelets, may provide data that enables estimation of platelet counts. However, because the ELISA method is time-consuming, the measurement of PDGF is not appropriate for quality assurance of A-PRF/CGF preparations prior to clinical use in case of on-site preparation. Therefore, other accurate methods that enable us to directly count platelets should be developed to assure the quality of A-PRF/CGF preparations.

## 4. Materials and Methods

### 4.1. PRP and PRGF Preparation

In a previous article [16], we literally compared PRP and other PRP derivatives and concisely described the difference of their characteristics.

As previously described [17,18,19], blood samples (~9.0 mL) were collected in the presence of acid citrate dextrose solution-A formulation (ACD-A; Terumo, Tokyo, Japan), an anti-coagulant, using plastic vacuum blood collection tubes (Neotube^®^; NIPRO, Osaka, Japan) equipped with 21-gauge needles from healthy, non-smoking volunteers (nine males; 28 to 71 years old). As listed in Table 1, to obtain the PRP (1st spin of the double-centrifugation protocol) and PRGF preparations, the blood samples were centrifuged using a KS-5000 centrifuge (Kubota, Tokyo, Japan) equipped with a swing rotor at 2480 rpm (1100× *g*) and 1800 rpm (580× *g*), respectively, at 25 °C for 8 min.

The study design and consent forms for all procedures performed within the study subjects were approved by the ethical committee for human subjects at Niigata University School of Medicine in accordance with the Helsinki Declaration of 1975 as revised in 2008.

### 4.2. Simulation of A-PRF and CGF Preparation in the Presence of the Anti-Coagulant

As described previously [13,20,21], blood samples (~9.0 mL) were collected with ACD-A, using conventional glass vacuum blood collection tubes (Vacutainer tube^®^; Becton, Dickinson and Company, Franklin Lakes, NJ, USA), from the same donors, and were immediately centrifuged using a Spectrafuge 6C^®^ centrifuge (Labnet International Inc., Edison, NJ, USA) equipped with an angle rotor or a Medifuge^®^ centrifugation system (Silfradent S. r. l., Santa Sofia, Italy) according to the protocol for preparation of A-PRF or CGF, respectively. The centrifugal conditions are listed in Table 1.

### 4.3. Preparation of A-PRF and CGF in the Absence of the Anti-Coagulant

For preparation of the A-PRF and CGF, the recommended glass vacuum blood collection tubes, A-PRF+^®^ (Jiangxi Fenglin Medical Technology Co. Ltd., Fengcheng, China) and Vacutainer tube^®^ were used, respectively. Blood samples were collected without the anti-coagulant and immediately centrifuged under the conditions listed in Table 1.

### 4.4. Determination of Blood Cell Counts

The number of blood cells in the initial whole blood and fractionated liquid samples were determined using an automated hematology analyzer (pocH-100iV diff; Sysmex, Kobe, Japan). First, RBCs, WBCs, and platelets were counted immediately after blood collection. Second, freshly prepared fractions in the presence of the anti-coagulant were immediately evaluated for blood cell count. To maximize the collection of platelets, the border between the RBC fraction and upper plasma fraction was established as 1 mm below the apparent border between these fractions (see Table 1).

To determine WBC and platelet counts in the absence of the anti-coagulant, after centrifugation, the supernatant serum fraction, if any, was collected first. Then, the resulting clot was removed, and the semi-clotted, or loosely-clotted, red thrombus was scrapped off, using dental tweezers, from the upper fibrin clot approximately 1 mm below the apparent border (Table 1, Figure 5). The resulting fibrin clot was then compressed using a PRF compressor [13] to squeeze the fibrin clot exudate. The RBC clot was minced with scissors and gently mixed by inverting the tube several times. Each fraction was subjected to cell counting using the hematology analyzer. Platelet and WBC counts in the fibrin clot were determined by subtracting those counts in the RBC, supernatant serum, and A-PRF/CGF exudate fractions from those counts in the anti-coagulant-free whole blood sample, as calculated according to the following Formula (1).

(PLTs/WBCs in fully clotted A-PRF/CGF preparations) = (PLTs/WBCs in liquid WB) − [(PLT/WBCs in semi-clotted RBC fraction) + (PLTs/WBCs in liquid serum) + (PLTs/WBCs in liquid exudate fraction)],(1)

To determine the numbers of WBCs and platelets attached to the inside wall of the tubes, after removing the clots and other liquid fractions, the tube was washed thoroughly with PBS three times, and tightly adherent WBCs and platelets were detached with 0.05% trypsin plus 0.53 mM EDTA (Wako Pure Chemicals, Osaka, Japan) with gentle agitation for 5 min. Cell suspensions were directly subjected to cell counting.

### 4.5. Scanning Electron Microscopy (SEM)

To detect platelets in the upper region of the red thrombus, the region below the border, which is indicated by a yellow dot-square in Figure 4, was dissected, washed in PBS three times, fixed with 2.5% glutaraldehyde, dehydrated with a series of ethanol and t-butanol washes, freeze-dried, and then examined by SEM (TM-1000, Hitachi, Tokyo, Japan) with an accelerating voltage of 15 kV, as described previously [22]. Aggregated platelets were microscopically examined, but not counted.

To observe platelet morphology on cell culture wares, the PRP was prepared as described previously [17,23], and the platelets were washed with a 10 mM Hepes–Tyrode buffer (pH 7.4), suspended in a Hepes–Tyrode buffer containing 100 ng/mL prostaglandin E1 (Cayman Chemical, Ann Arbor, MI, USA), and stored while gently stirring with a rotator at ambient temperature until used, usually within 2 days. Platelets were placed on plastic dishes, lids of plastic dishes, or glass coverslips for 10 min at 37 °C. Then, platelets were fixed and prepared for examination by SEM.

### 4.6. Statistical Analysis

The data are reported as the mean value ± standard deviation (S.D.). For multi-group comparisons, statistical analyses were performed to compare the mean values by one-way analysis of variance (ANOVA) (SigmaPlot 12.5; Systat Software, Inc., San Jose, CA, USA). When the data did not pass the normality test, Dunn’s (for platelet counts in the RBC fraction) or Tukey’s multiple comparison tests were performed. *p*-values < 0.05 were considered significant.

## 5. Conclusions

Significant numbers of platelets are present in the RBC portion of fractionated whole blood at greater levels than expected, especially after centrifugation with a higher centrifugal force. In the absence of the anti-coagulant, platelets are aggregated on fibrin meshwork of the red thrombus and cannot be easily released for counting. Therefore, it is suggested that the current subtraction method is not appropriate for the determination of platelet counts in clotted A-PRF/CGF preparations. An accurate, direct, simplified method should be developed immediately to help the quality assurance of A-PRF/CGF preparations for clinical use.

## Figures and Tables

**Table 1:** Centrifugal conditions for preparation of four platelet concentrate types and the resulting fractions.

Centrifugation	PRP	PRGF	A-PRF	CGF
Force (g)	1100	580	200	692
				547
				592
				855
Duration (min)	8	8	8	2
				4
				4
				3
Appearance of ACD-A-contained blood samples